# Targeting the *Pseudomonas* quinolone signal quorum sensing system for the discovery of novel anti-infective pathoblockers

**DOI:** 10.3762/bjoc.14.241

**Published:** 2018-10-15

**Authors:** Christian Schütz, Martin Empting

**Affiliations:** 1 Helmholtz-Institute for Pharmaceutical Research Saarland (HIPS) - Helmholtz Centre for Infection Research (HZI), Department of Drug Design and Optimization (DDOP), Campus E8.1, 66123 Saarbrücken, Germany; 2 Department of Pharmacy, Saarland University, Campus E8.1, 66123 Saarbrücken, Germany; 3 German Centre for Infection Research (DZIF), Partner Site Hannover-Braunschweig, Saarbrücken, Germany

**Keywords:** anti-infectives, pathoblockers, PQS, *Pseudomonas aeruginosa*, quorum sensing

## Abstract

The Gram-negative opportunistic pathogen *Pseudomonas aeruginosa* causes severe nosocomial infections. It uses quorum sensing (QS) to regulate and coordinate population-wide group behaviours in the infection process like concerted secretion of virulence factors. One very important signalling network is the *Pseudomonas* quinolone signal (PQS) QS. With the aim to devise novel and innovative anti-infectives, inhibitors have been designed to address the various potential drug targets present within pqs QS. These range from enzymes within the biosynthesis cascade of the signal molecules PqsABCDE to the receptor of these autoinducers PqsR (MvfR). This review shortly introduces *P. aeruginosa* and its pathogenicity traits regulated by the pqs system and highlights the published drug discovery efforts providing insights into the compound binding modes if available. Furthermore, suitability of the individual targets for pathoblocker design is discussed.

## Introduction

In recent years, attempts to raise public awareness on antimicrobial resistance (AMR) and the large threat that it poses towards modern health standards have been made [1]. It is an alarming notion that at an increasing rate of available treatment options proves ineffective in eradicating bacterial infections [2]. Especially in the case of Gram-negative bacteria, an urgent need for novel medicines has been identified while the pipeline of drug candidates is literally running dry and a desirable renaissance of the golden age of antibiotic drug research in ‘big pharma’ is currently not to be seen on the horizon [3–4]. Nevertheless, some innovative strategies to be explored for their clinical applicability in combating bacterial infections have been devised in the last decades mostly driven by academic research [5–7]. In contrast to addressing classical antibiotic drug targets involved in vital processes of the bacterial cell, ‘antivirulence’ strategies aim at abolishing pathogenic features without affecting cell viability, providing the basis for a lower drug-induced selection pressure [5,8–9]. Hence, a reduced rate of resistance development is expected [9]. A clinical proof-of-concept for this unconventional strategy has been provided recently by the approval of the toxin-neutralizing therapeutic antibody bezlotoxumab, which is henceforth in clinical use for pre-emptive treatment of recurring clostridial infections [10]. So, the potential of active principles, which do not kill the bacteria through bactericidal or bacteriostatic effects, but mediate their effect through pathogen-specific action on virulence mechanisms, has been unveiled. This short review focuses on the current knowledge of one particular antivirulence strategy against the important pathogen *Pseudomonas aeruginosa*, which is based on the disruption of the *Pseudomonas* quinolone signal quorum sensing system (pqs QS).

## Review

### Antimicrobial resistance and clinical relevance of *Pseudomonas aeruginosa*

*P. aeruginosa* is one of the threatening ESKAPE pathogens and has regularly been attributed with the label ‘superbug’ [11]. In 2017, the World Health Organization (WHO) has published a priority list for pathogens with urgent need for novel treatment options and carbapenem-resistant *P. aeruginosa* was ranked in the highest category ‘critical’ [12]. One of the main problems we face regarding this Gram-negative bacterium is that it shows a prominent ability to resist antibiotic treatment via several mechanisms. First and foremost, it possesses an intrinsic resistance to many antibiotics because of the low permeability of its cell wall and due to the action of a number of efflux pumps as well as β-lactamases. Efflux pumps in particular are nifty molecular machineries consisting of several protein components, which in total span from the inner to the outer side of the cell membrane. Their function is to expel a wide range of xenobiotics, among them antibiotics from the cephalosporin, carbapenem, fluroquinolone and aminoglycoside classes [13]. Through this mechanism, these drugs cannot reach their intracellular targets rendering them ineffective. β-Lactamases, on the other hand, act specifically on compounds which carry the eponymous cyclic moiety as the activity-driving motif and their genes are found to be encoded on the chromosomes of many *P. aeruginosa* strains. Hence, these antibiotic-inactivating enzymes provide resistance against penicillins and cephalosporins [14].

In addition to these intrinsic capabilities, *P. aeruginosa* is able to acquire resistances toward antibiotics it has come in contact with. These acquired resistances can be the result of spontaneous mutations in genes encoding for the target protein. For example, certain mutational changes within DNA gyrase will lead to lowered susceptibility for fluoroquinolones [15]. Other examples are mutants leading to efflux pump overexpression [15]. If the resistance determinant is located on a transferable plasmid, it can be efficiently spread among bacteria via horizontal gene transfer, which is probably the most frequent mechanism for the development of acquired resistances [15]. In these cases, the resistance determinant is inheritable and passed over to the next generation of bacteria.

Furthermore, a mechanism has been discovered, which is referred to as adaptive resistance and describes the observation that a persistent environmental stimulus can induce non-mutational resistances [15]. Under continuous treatment regimes, the antibiotic itself can of course be the stimulus. But, nutrient deprivation, pH, anaerobiosis, as well as biocides, polyamines, cations and carbon sources could also act as external triggers leading to adaptive resistance. The common effect of these stimuli seems to be an alteration in expression patterns ultimately impacting, e.g., efflux pump or enzymatic activity, as well as cell envelope properties or biofilm formation [15].

All the mechanisms described above help to explain the notion that established chronic *P. aeruginosa* infections are notoriously difficult to eradicate. This ubiquitous opportunistic pathogen is able to cause infections basically in every niche of the human body where it finds enough moisture [16]. Common sites of infection are the respiratory and urinary tracts, the eye and wounds, e.g., those resulting from burn injuries [17]. These occur frequently in hospitalized and especially immunocompromised individuals. Patients with chronic lung diseases like cystic fibrosis (CF) or bronchiectasis have a poor prognosis when *P. aeruginosa* colonisation is detected, as this is usually associated with loss of lung function, morbidity, and mortality [18]. In 2013, it has been estimated, that by the age of eighteen 80% of the CF patients are *Pseudomonas* positive. Recently, evidence has been provided that this ratio is reducing [19]. Nevertheless, with progression of age the majority of CF patients will become chronically infected with *P. aeruginosa* and this is still the major cause of death associated with this genetic disorder [20]. Importantly, it has been described that the amount of quinolone-based quorum sensing (pqs QS; vide infra) in those patients correlates with a negative prognosis and might function as a possible biomarker for the severity of the infection [21].

### Quroum sensing (QS)

**Figure 1:**
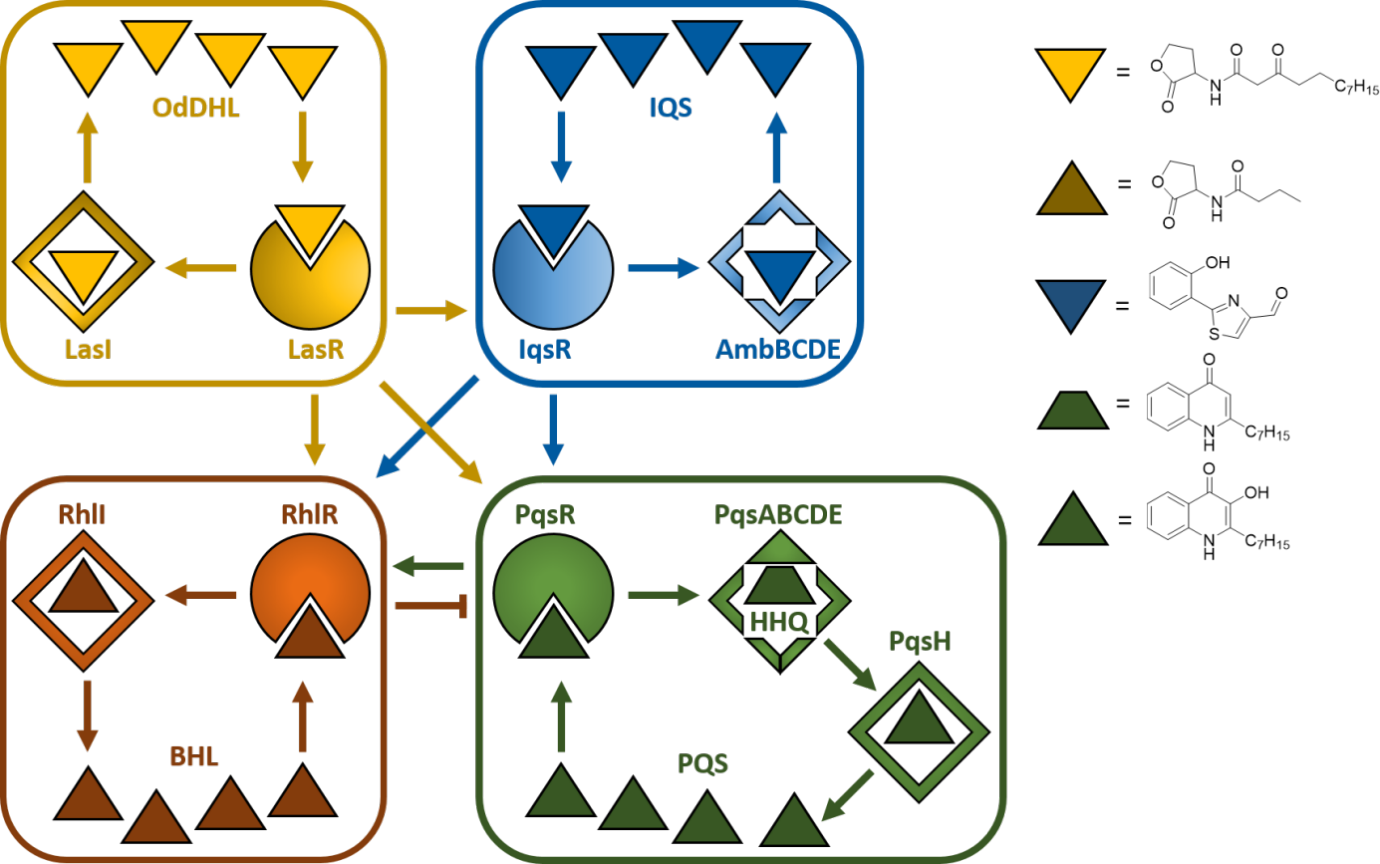
The four quorum sensing systems in *P. aeruginosa las, iqs*, *rhl*, and *pqs*. Abbreviations: OdDHL, *N*-(3-oxododecanoyl) homoserine lactone; IQS, integrating quorum sensing signal; BHL, *N*-butyryl-L-homoserine lactone; PQS, *Pseudomonas* quinolone signal. Positive control is represented by arrows, negative control by blunted arrow.

In general, the term quorum sensing describes a population-density-dependent cell-to-cell communication system making use of small diffusible molecules as signalling agents. By this means, pathogenic bacteria can coordinate population-wide changes to expression patterns and regulate concerted group behaviours important in the infection process. Critical pathogenicity traits like the production of virulence factors or biofilm formation are under the control of these systems. Actually, title pathogen makes use of four intertwined QS systems, referred to as las, rhl, pqs, and iqs [22]. These subsystems influence each other establishing an intricate regulatory network with compensatory mechanisms ensuring environmental adaptability and fine-tuned control of associated virulence genes (Figure 1). All four have been studied in the pursuit of quorum sensing inhibitors (QSI) to be used as blockers of *P. aeruginosa* pathogenicity [11,23].

Typically, a QS system of Gram-negatives consists of a transcription regulator, the signal molecules and one or several enzymes involved in the synthesis of the latter. The regulator usually controls the transcription of the biosynthetic enzymes and also functions as a receptor for the signal molecules themselves. As these are actually autoinducers (AIs) and, hence, have an agonistic activity toward their receptor, a positive feedback loop is created. In *P. aeruginosa* three different chemotypes of AIs have been identified, to date: alkyl homoserine lactones (AHLs) used by the las and rhl systems, alkylquinolones (AQs) used by the pqs system and 2-(2-hydroxyphenyl)thiazole-4-carbaldehyde used by the iqs system (Figure 1). Strategies addressing las and rhl have been reviewed elsewhere [5,11], while to date one study on iqs inhibition has been reported [23]. Many drug discovery efforts towards effective pathoblockers have been published based on the design and optimisation of pqs targeting QSI, which is the topic of this review.

### The biosynthetic cascade of the pqs QS system

**Figure 2:**
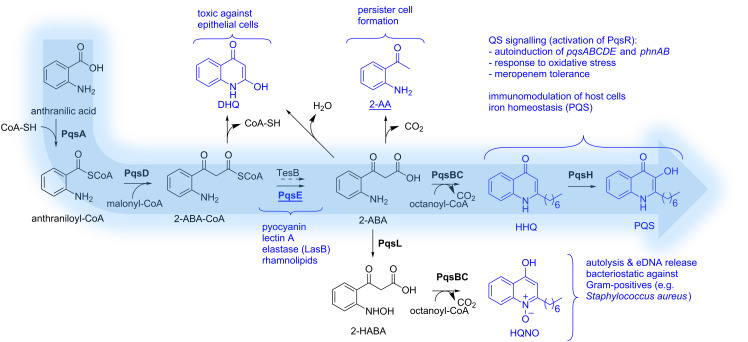
Schematic overview of the PQS biosynthesis and involvement of related metabolites and PqsE in virulence. Effector molecules are highlighted in blue. Enzymes are given in bold. Abbreviations: CoA, coenzyme A; 2-ABA-CoA, 2’-aminobenzoylacetyl-CoA; 2-ABA, 2’-aminobenzoylacetate; DHQ, dihydroxyquinoline; 2-AA, 2’-aminoacetophenone; 2-HABA, 2’-hydroxylaminobenzoylacetate; HHQ, 2-heptyl-4-(1*H*)-quinolone; HQNO, 4-hydroxy-2-heptylquinoline-*N-*oxide; PQS, *Pseudomonas* quinolone signal.

PQS is the abbreviation for *Pseudomonas* quinolone signal and actually refers to the signal molecule 2-heptyl-3-hydroxyquinolin-4(1*H*)-one (Figure 2). This quinolone-based AI and its biosynthetic precursor HHQ (2-heptylquinolin-4(1*H*)-one) are ligands of a transcription factor called ‘multiple virulence factor regulator’ (MvfR), also referred to as PqsR. Through interaction with this receptor, HHQ and PQS induce the transcription of a variety of genes including their own biosynthetic enzyme cascade (PqsABCDE). Together with PqsH and PqsL, which are under the control of LasR from the las QS system, these enzymes manage to build up PQS and related molecules from anthranilic acid (Figure 2). This initial building block can be provided either through the kynurenine pathway starting from tryptophan or by anthranilate synthases from the PqsR-controlled phnAB operon starting using chorismic acid as a source [24]. Either way, the ligase PqsA starts PQS synthesis by condensing anthranilic acid with coenzyme A [25]. The resulting activated thioester (anthraniloyl-CoA) is then transferred to an active-site cysteine of the β-ketoacyl-ACP synthase III (FabH)-type enzyme PqsD [26–27]. Subsequently, another CoA-activated substrate comes into play. In analogy to fatty acid synthesis, malonyl-CoA is reacted with the enzyme-bound thioester to yield 2-aminobenzoylacetyl-CoA (2-ABA-CoA) under decarboxylation [28–29]. In a next step, the pathway-specific thioesterase PqsE generates 2-aminobenzoylacetate (2-ABA) [29]. It has been shown, that also the broad-specificity thioesterase TesB present in *P. aeruginosa* can catalyse this reaction [29]. The quinolone core is formed by action of the heterodimeric complex PqsBC. This time, CoA-activated octanoic acid is used to preload an active-site cysteine of PqsC with the fatty acid via a thioester linkage [30–31]. The previously produced 2-ABA is then consumed to from HHQ under decarboxylative condensation [30]. Finally, PQS is produced through hydroxylation of position 3 by the NADH-dependent flavin mono-oxygenase PqsH [32].

This biosynthetic cascade is also responsible for the generation of the pqs-related metabolites DHQ, 2-AA, and HQNO as well as other AQs having different lengths of the alkyl chain [29–30]. Aforementioned enzyme PqsL is needed for the production of HQNO, as it delivers the *N*-oxidised substrate 2-HABA for PqsBC-mediated condensation with octanoyl-CoA analogous to HHQ biosynthesis [27].

### PQS-mediated pathogenicity traits and molecular targets

*P. aeruginosa* makes use of an arsenal of virulence factors and other pathogenicity traits to overwhelm and colonise the host in the infection process [5] and pqs QS plays a crucial role in the regulation of many of those. It is astonishing, that expression of 182 genes is altered in response to exogenous PQS [33]. Evidence has been gathered, that these effects are mediated either through direct PqsR-dependent action or by PqsR-independent mechanisms, which are most likely due to the iron-chelating as well as antioxidant properties of PQS [33]. Furthermore, it has been unravelled that the thioesterase PqsE, whose biosynthetic function is dispensable due to the presence of alternative thioesterases in *P. aeruginosa*, is actually also a major effector molecule of *pqs* QS [33]. Via a yet unknown mechanism, this enzyme regulates 145 genes, while only 30 of these overlap with the PQS regulon. It seems that these two are the main mediators of *pqs* QS response. In terms of pathogenicity traits, they are involved in the regulation of genes encoding for enzymes responsible for phenazine biosynthesis (pyocyanin production), hydrogen cyanide synthesis, Lectins LecA and LecB and additional genes involved in biofilm formation, enzymes for rhamnolipid synthesis, a Resistance-Nodulation-Cell division (RND) efflux pump encoded by *mexGHI*-*opmD* operon, components of Type 3 and Type 6 secretion as well as the exotoxin ExoS, and siderophore synthases [33].

In addition to virulence regulation, some remarkable secondary effects have been attributed to the PQS molecule [34]. This autoinducer has been described to mediate iron acquisition, cytotoxicity, outer-membrane vesicle biogenesis, and to exert host immune modulatory effects [34–35]. Interestingly, PQS as well as HHQ are able to interfere with nuclear transcription factor-κB and hypoxia-inducible factor 1 (HIF-1) signaling pathways and, thus, down-regulate host innate immune systems [36–37]. Other PQS-related metabolites have been shown to have additional effects. HQNO, for example, induces autolysis and release of extracellular DNA thereby promoting biofilm formation and increasing meropenem tolerance [38]. HQNO acts through inhibition of complex III in the respiratory chain of bacteria and mitochondria of eukaryotes and, hence, it can be considered a general cytotoxic agent. DHQ, a shunt product of the PQS biosynthetic pathway, is important for *P. aeruginosa* virulence in a *Caenorhabditis elegans* model and also excerts a growth inhibitory effect on epithelial cells [26,39]. Finally, 2-AA has been described to be important for persister cell formation, a very important tolerance mechanism against antibiotic treatment [40].

Among the virulence factors which are directly or indirectly controlled by pqs QS, pyocyanin is one of the most prominent. This redox-active pigment is responsible for the greenish-blueish colour of *P. aeruginosa* cultures. It seems that generation of reactive oxygen species is a major mechanism of pyocyanin cytotoxicity [41]. This tricyclic compound is known to induce apoptosis in neutrophils, but also to enhance neutrophil extracellular trap formation [42–43]. Both mechanisms impair neutrophil-mediated host defenses. Additionally, it has been hypothesised that pyocyanin functions as an extracellular electron shuttle, contributing to redox homeostasis of *P. aeruginosa* cells in biofilms with anaerobic conditions [44].

Due to these important virulence mechanisms, which are under direct or indirect control of pqs QS, targeting this master regulatory system with small molecular compounds, thereby blocking *P. aeruginosa* pathogenicity, is very attractive. However, this complex network of biosynthetic pathways and effector molecules renders selection of the perfect point for intervention difficult. Due to their rather peripheral role in AQ synthesis, PqsH and PqsL, have not been of significant interest for QSI discovery to date. However, all enzymes of the primary biosynthetic cascade pqsA–E as well as the signal molecule receptor PqsR might be valuable drug targets. Also, agents capable of modulating more than one target could be of interest. The question is, which of these targets and/or combinations asserts the most relevant virulence-attenuated phenotype after QSI treatment.

### PqsA inhibitors

#### Anthranilic acid analogues

**Figure 3:**
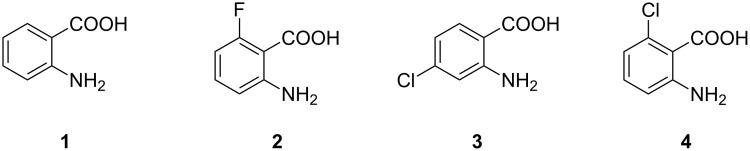
Anthranilic acid (**1**) and derivatives thereof (**2**–**4**).

Since anthranilic acid (**1**) serves as a PqsA substrate, the first compound reported to inhibit PqsA is 6-FABA (**2**, Figure 3), which was able to block this enzyme and successfully suppressed the production of DHQ in PA14 strains at a rather high concentration of 1.5 mM. Moreover, it was shown that 6-FABA had no impact on the bacterial growth. Lépine et al. suggested that **2** competitively occupies the active site of PqsA [45] and therefore serves as a substrate analogue of AA (**1**). It was stated that the introduction of electron-withdrawing substituents could prevent activation of the carbonyl group as a CoA-ester.

**Figure 4:**
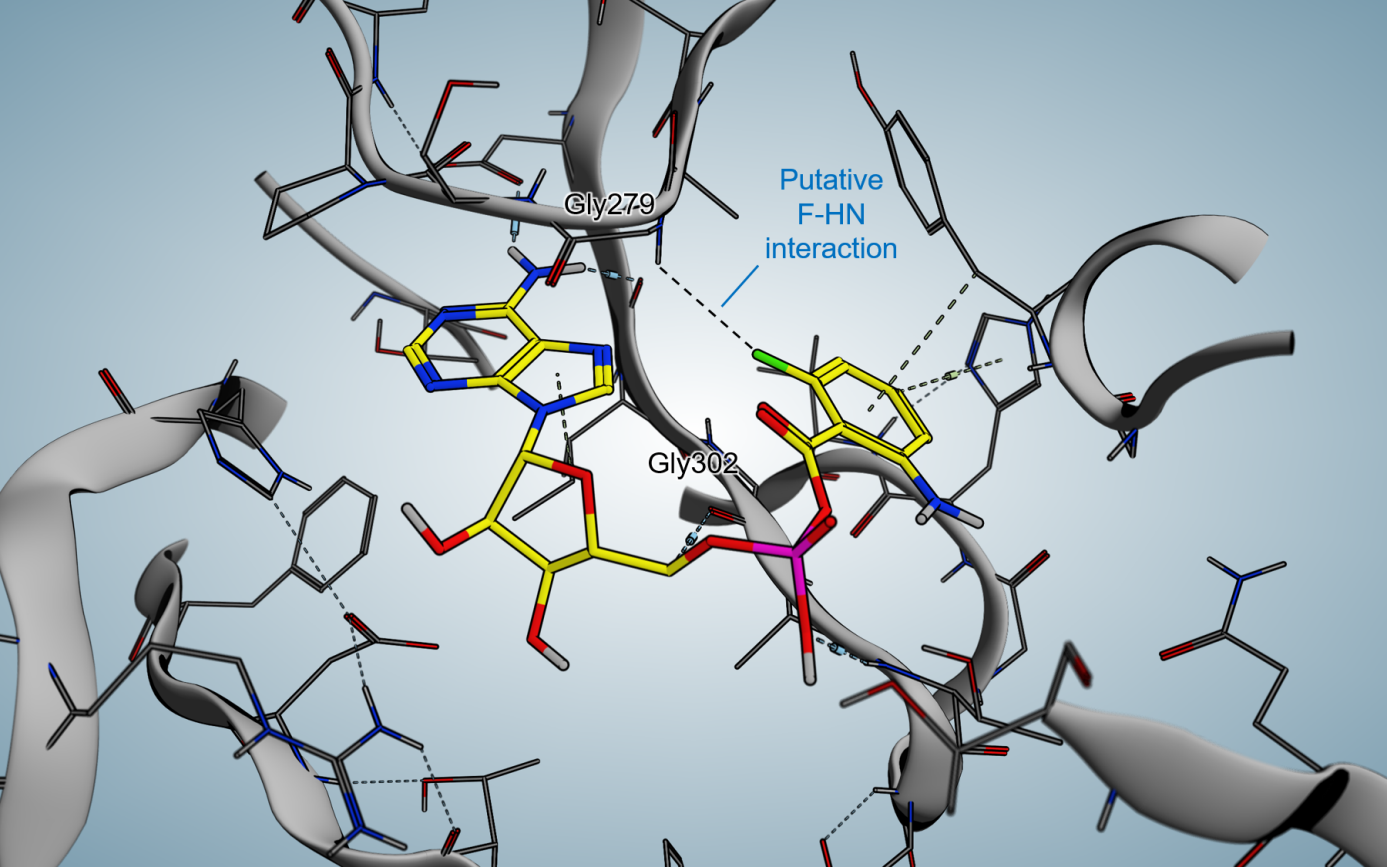
Crystal structure of 6-FABA-AMP in complex with PqsA.

In 2017, Witzgall et al. were able to co-crystallize 6-FABA-AMP within the *N*-terminal domain of PqsA (Figure 4) [46].

Key interactions involve a water-mediated hydrogen bond between the amino function of the compound and Q162, as in anthraniloyl-AMP. The reason why the fluorinated anthraniloyl-AMP shows good affinity is the formation of a hydrogen bond of the fluorine with the G279 backbone amide hydrogen and furthermore an interaction with the N7 position of the adenine moiety. Additionally a very typical fluorine/main-chain interaction with G302 could be observed.

Various halogenated derivatives of AA could also reduce HHQ and PQS levels. Especially 4- and 6-CABA (**3**, **4**) showed promising results in the suppression of signal molecules as well as in an in vivo mouse survival model at a concentration of 1.5 mM [47].

#### Anthraniloyl-AMP mimetics

**Figure 5:**
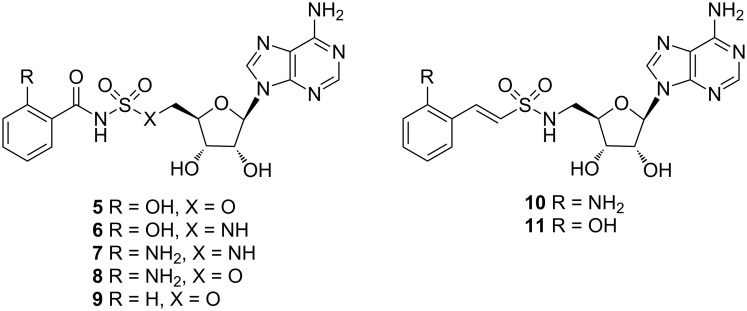
Structures of substrate mimetic PqsA inhibitors.

More recently, Ji et al. published two classes of sulfonyladenosine inhibitors, more precisely the sulfamate/sulfamide inhibitors **5**–**9** and the vinyl sulfonamide inhibitors **10** and **11** (Figure 5). While the latter showed very low affinity for the protein, the former displayed *K*_i_ values between 88 nM for compound **7** and 420 nM for compound **9**. Despite these promising results, the designed molecules were not able to reduce the signal molecules HHQ and PQS at satisfactory levels (300 µM < IC_50_ < 880 µM).

A plausible reason for this outcome might be low cell penetration and/or efflux pump mechanisms, which was supported by compound accumulation studies [48].

### PqsD inhibitors

**Figure 6:**
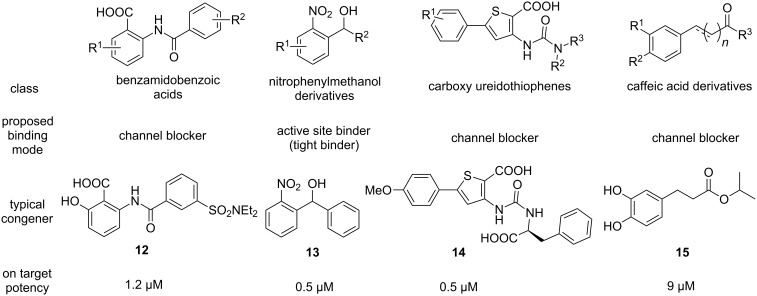
Structures and characteristics of prominent classes of PqsD inhibitors.

PqsD, the second enzyme in the biosynthetic cascade, has been studied intensely by the Hartmann group. Several design strategies have been pursued leading to diverse structural classes of inhibitors (Figure 6). Unfortunately, for none of these compounds an X-ray structure in complex with PqsD has been reported although the apoenzyme as well as a substrate-bound form has been successfully crystallized [49]. Using these coordinates, employing in silico methods allowed proposing plausible binding poses for prototypic analogues of the respective structural classes.

The first reported inhibitors of PqsD were 2-benzamidobenzoic acids [50]. In a pioneering study on the biosynthetic function of this β-ketoacyl-ACP synthase III (FabH)-type enzyme, known blockers of FabH were described to also inhibit this related target [50]. The claim that PqsD is directly responsible for HHQ production by using anthraniloyl-CoA and β-ketodecanoate as substrates, had to be revised later to also include PqsE and PqsBC as participants in AQ biosynthesis in *P. aeruginosa* (vide supra) [27]. Nevertheless, this conversion is indeed catalysed by PqsD in vitro and was successfully exploited for devising a valuable assay, which served as an SAR driver for most of the literature-known PqsD-directed projects. Further benzamidobenzoic acid derivatives were explored for their efficacy and a binding mode was proposed based on SPR- and STD-NMR-assisted docking [51]. These inhibitors appeared to bind in the substrate channel in a slightly remote position from the active site cysteine and, hence, termed channel blockers [51]. Optimised hits exhibited a potency in the single-digit micromolar range (**12**, Figure 6). However, it has been found that similar compounds also showed activity against RNA polymerase, a popular target for the development of new antibiotics [52–53]. Hitting such a target would jeopardise the principle of pathoblockers, which should only disarm the bacteria and not kill them. Hence, a follow-up study on PqsD/RNAP selectivity was conducted providing insights into motifs granting selective PqsD inhibition [52].

In a ligand-based approach, nitrophenylmethanol derivatives were identified as fragment-sized inhibitors of PqsD. Initially, these compounds where designed as transition state analogues mimicking the tetrahedral reaction intermediate between PqsD and anthraniloyl-CoA [54]. Upon simplification and rigidification through reduction in size as well as removal of rotatable bonds inhibitor **13** was obtained carrying the characteristic secondary alcohol of this class. Notably, both enantiomers of **13** show similar potency, but different thermodynamic profiles as measured via isothermal titration calorimetry (ITC) [55]. Despite its low molecular weight, **13** showed tight-binding kinetics and was able to reduce production of HHQ, as well as PQS. Furthermore, it was capable of attenuating biofilm production [54]. All the information gathered via site-directed mutagenesis combined with thermodynamic profiling, as well as surface plasmon resonance (SPR) experiments with and without covalent active site blockade, corroborated that the nitrophenylmethanol class directly binds to the active site near the reactive cysteine of PqsD [55]. This is in line with the initial transition state analogue design principle. Further structural exploration of this class showed that this fragment-like size helps to retain cellular activity [56]. While fragment growing could increase target activity to the nanomolar range, a complete loss of efficacy in the *P. aeruginosa* quorum quenching assays was observed [56]. This highlights a notable issue when addressing intracellular targets of this Gram-negative bacterium, as permeating the outer and inner membrane while escaping efflux and enzymatic deactivation may represent a true challenge.

**Figure 7:**
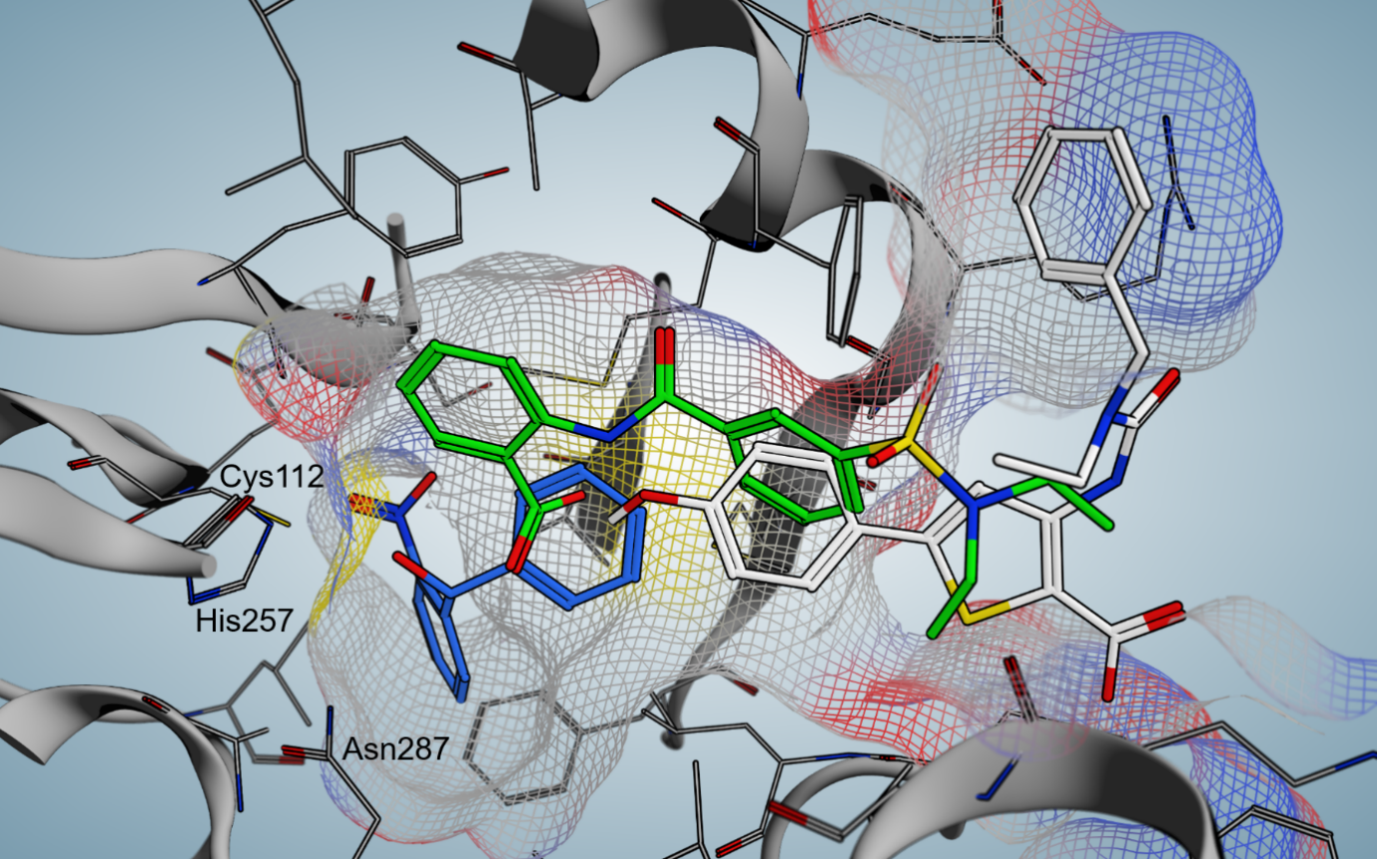
Comparison of docking poses of three prototypic PqsD inhibitors: benzamidobenzoic acid derivative **12** (green), nitrophenylmethanol derivative **13** (blue), carboxy ureidothiophene derivative **14** (white). Active site residues are labelled and the surface of the substrate tunnel is indicated by a mesh.

The elucidation of the binding mode of the nitrophenylmethanol class was then exploited to gain insights into the interaction profile of another chemotype of PqsD inhibitors – the ureidothiophenes (Figure 6) [57–58]. An initial hit showing activity against the enzyme in the single-digit micromolar range was studied using a tailor-made SPR experiment including truncated and elongated derivatives as well as nitrophenylmethanol-based active-site blockers of different size as competitors. These experiments combined with molecular docking (Figure 7) led to the postulation of a plausible binding pose characterising the ureidothiophenes as channel blockers. This model was successfully used for further optimisation attempts and nanomolar potency in the enzyme assay was achieved (**14**, Figure 6) [57–58]. Notably, a nucleophilic warhead could be introduced specifically reacting with the active-site cysteine through elongation into the substrate tunnel [57]. The binding models of the ureidothiophene and nitrophenylmethanol classes even allowed for the generation of a merged inhibitor [58]. One major liability of this class, however, was the general inefficacy in whole cell assays, which could not be improved, even through the attachment of a cell-penetrating peptide sequence [58].

One additional class, which did show cellular activity, was based on a catechol scaffold [59]. In analogy to the successful discovery of PqsD inhibitors starting from known FabH-targeting compounds (vide supra), ligands of another enzyme with high similarity to the signal molecule synthase were investigated. Here, substrates of chalcone synthase CHS2 from *Medicago sativa* were tested for their ability to block PqsD function. Indeed, caffeic acid analogues, such as **15**, were identified as hits and further characterized as channel blockers as described before [59].

**Figure 8:**
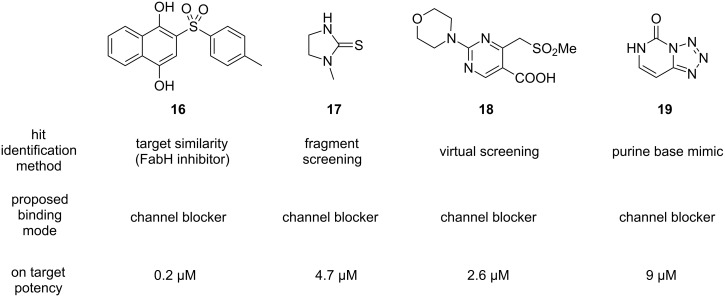
Structures and characteristics of hits against PqsD identified through different methods.

Further interesting starting points for the discovery of PqsD inhibitors have been provided by a dedicated screening campaign involving fragment-based hit discovery, in silico screening and a similarity-guided approach starting from FabH inhibitors [60]. The most potent hit **16** of this study showed activity in the nanomolar range (Figure 8). Furthermore, a tetrazolopyrimidinone scaffold **19** has been reported to inhibit PqsD through a putative blockade of the CoA binding site [61].

**Figure 9:**
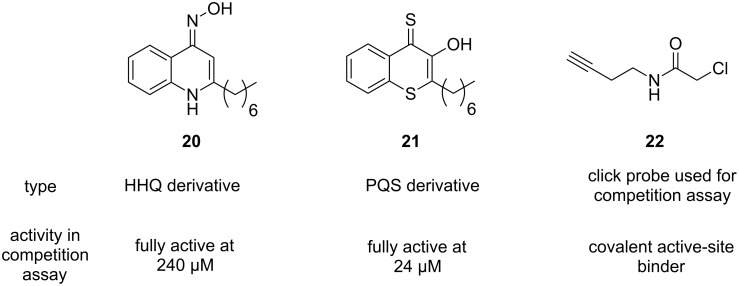
HHQ and PQS analogues as PqsD inhibitors and chemical probe used for screening.

The Böttcher group used a library of HHQ as well as PQS analogues to screen for PqsD inhibition [62]. To this end, a novel competition assay employing ‘clickable’ active-site-labelling probes was developed. These compounds contain terminal alkyne moieties, which can be exploited for straightforward decoration via copper(I)-catalyzed alkyne–azide cycloaddition (CuAAC), the prototypic click reaction. This facilitated the discovery of novel PqsD-targeting compounds through CuAAC-mediated conjugation of a fluorescent dye (Figure 9) [62].

**Figure 10:**
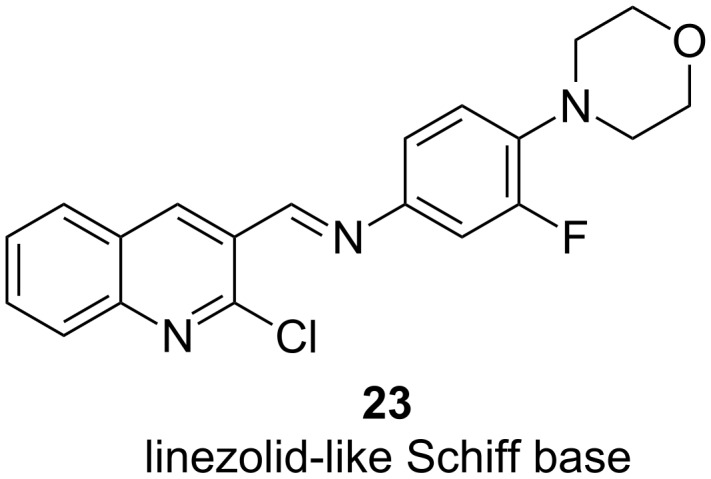
Structure of PqsD-targeting biofilm inhibitor derived from linezolid.

Finally, Sangshetti et al. reported the discovery of linezolid-like Schiff bases, which showed promising anti-biofilm activity in the double-digit micromolar range [63]. Notably, their potency in attenuating biofilm formation was more pronounced than ciprofloxacin and linezolid itself. A docking study suggested PqsD to be the target of these compounds like **23** (Figure 10), although this remains speculative.

### PqsE inhibitors

The pathway-specific thioesterase PqsE is not only responsible for hydrolyzing 2-ABA-CoA to form 2-ABA, but moreover also regulates bacterial virulence [29]. It has been shown that PqsE is a key effector of the *pqs* system and required for full *P. aeruginosa* virulence. One of its most prominent functions is the upregulation of pyocyanin, which is mediated through the *rhl* system. Notably, PqsE can still exert its function in absence of an active *pqs* QS [64–65]. Its important role in virulence regulation renders this enzyme an attractive target for pathoblockers.

**Figure 11:**
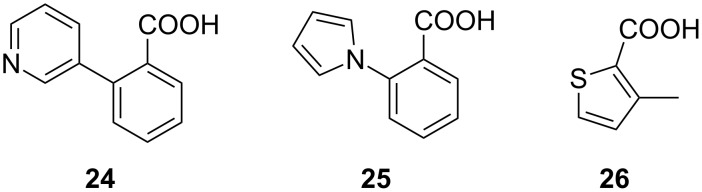
Fragment-based PqsE-inhibitors **24**–**26**.

In 2016, Zender et al. reported their attempt to inhibit PqsE through fragment-based screening. In order to block the thioesterase activity of the enzyme, a library of 500 fragments was screened via differential scanning fluorimetry (DSF) and the hit fragments **24**–**26** (Figure 11) were further validated using isothermal titration calorimetry (ITC) [66]. Binding to PqsE could be confirmed with *K*_D_ values of 0.9 ± 0.3 µM for **24** and 19.6 ± 3.7 µM for **26**.

The highly enthalpy-driven binding indicates a specific noncovalent interaction of **24** to the protein. To further investigate the binding mode of the hit fragments in the protein crystallization experiments were performed. Since the native substrate 2-ABA-CoA shows a short half-life, the reaction product 2-ABA was used as a surrogate to compare its binding mode with that of the hit fragments.

**Figure 12:**
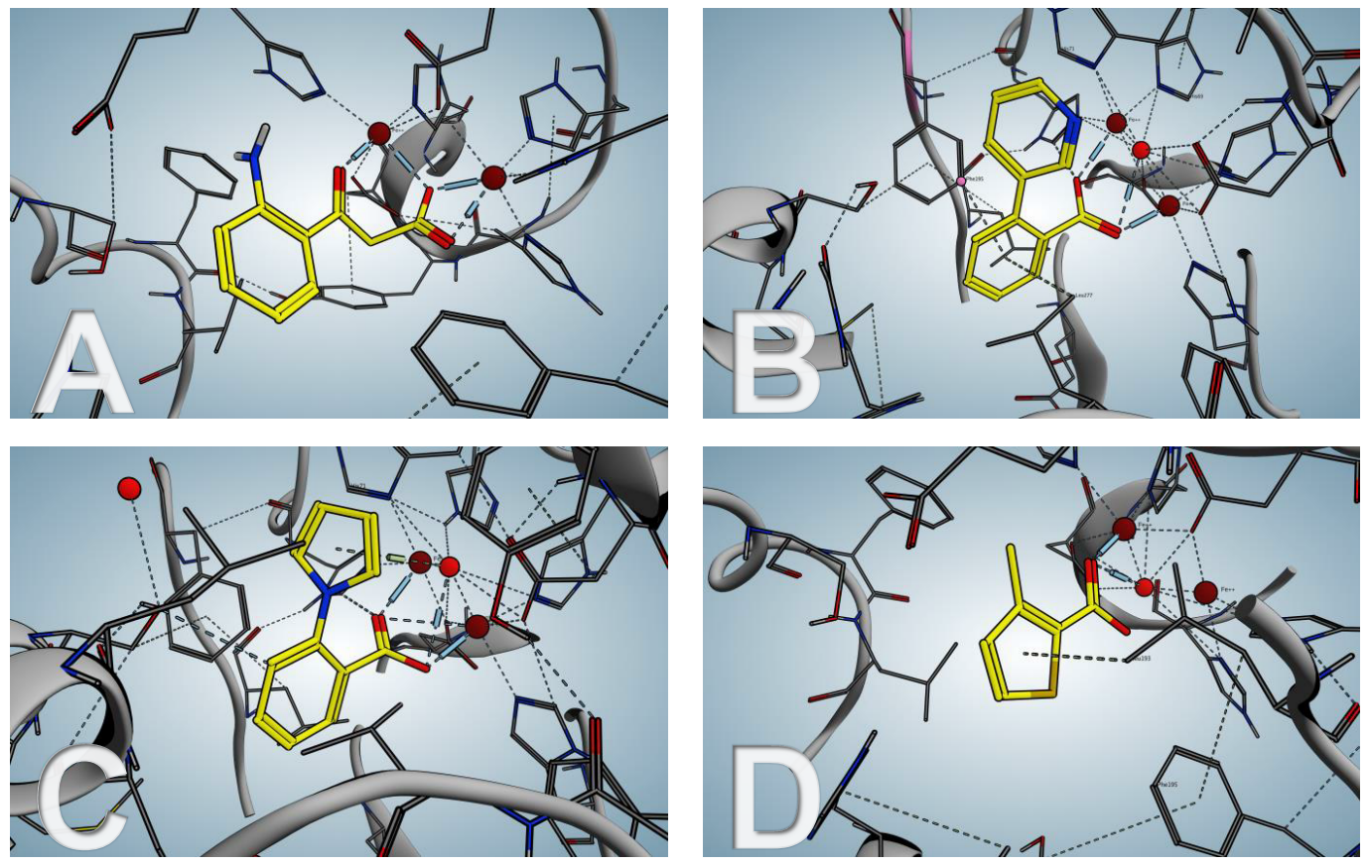
PqsE co-crystal structures. (A) native product 2-ABA; (B–D) hit fragments **24**–**26**.

Even though the screening hits occupy the same binding site as the native cleavage product 2-ABA, the binding mode is different. The fragments bridge the two metal atoms in the binuclear active center via a water molecule in contrast to 2-ABA, where the carboxylate occupies this position (Figure 12). Moreover both, 2-ABA and the ligands **24**–**26** are stabilized by hydrophobic interactions. Additionally, compounds **24** and **25** are interacting with a histidine (His71) sidechain via π-stacking. For the thiophene-containing fragment **26** a π–π interaction of the sulfur with Phe195 can be observed.

In vitro evaluation was performed using a combined PqsDE assay due to the aforementioned instability of 2-ABA-CoA which in this scenario is generated in situ from anthraniloyl-CoA via PqsD-mediated condensation with malonyl-CoA. The hit fragments were able to block the thioesterase in the micromolar range (e.g., IC_50_ (**24**) = 25 ± 4 µM). When assessing the hits on *Pseudomonas* cultures, thioesterase inhibition remained, whereas none of the compounds had any impact on pyocyanin production at a concentration of 500 µM. This means that the regulatory function of PqsE is not linked to its hydrolase function. Since the regulatory function of the enzyme is not associated to its active site, it was hypothesized that it might be involved in a macromolecule–macromolecule interaction, e.g., protein–protein or protein–DNA/RNA interaction, while the exact molecular mechanism remains unclear. Even though Zender et al*.* were not able to attenuate PA virulence via blockage of PqsE, important new insights on this target were made. The discovery that pathoblockers targeting PqsE assumedly may not need to target the active site of the enzyme, but rather a different pocket or surface. To this end, further research on the exact molecular mechanism of the regulatory activity of PqsE is needed.

### PqsBC

The small molecule 2-AA (**27**), which is also a secondary metabolite generated in the AQ biosynthesis pathway, was reported to inhibit PqsBC [31]. In a PqsBC-based biochemical assay it showed an IC_50_ in the micromolar range and was proven to reduce virulence in an acute mouse infection model [67].

**Figure 13:**
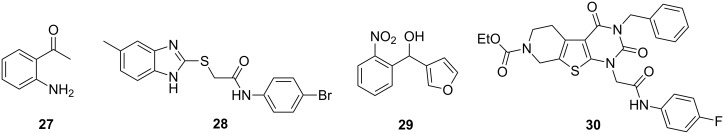
Structurally diverse PqsBC-inhibitors **27**–**30**.

In 2017, Maura et al. synthesized inhibitors based on a benzimidazole scaffold (Figure 13) [68]. Starting from a PqsR inhibitor, changes of the electronic properties on the benzimidazole by introducing an electron-donating group led to a higher PqsBC inhibitory activity, while decreasing the affinity to PqsR (compound **28**).

Nevertheless, it was shown that blockage of PqsBC leads to a reduction of HHQ but an accumulation of DHQ, which is reported to be toxic for epithelial cells, and 2-AA, which is involved in formation of persister cells [69]. In the same work, compounds **29** and **30** were evaluated. Compound **29** was first reported in a study aiming at the design of PqsD inhibitors, showing only very weak activity against this target. However, it showed surprisingly good effects on signal molecule production in cell-based assays. Later it was found that this compound actually gains its cellular activity through inhibition of PqsBC [56,70]. As already expected from previous results these compounds also showed a strong increase in 2-AA and DHQ production, while not affecting the overall production of AQ’s.

### PqsR

In 2017 Kamal et al. investigated the structure–functionality relationship of compounds targeting PqsR. They differentiated between agonists, neutral agonists, inverse agonists and agonistic/antagonistic mixed profile compounds. It was shown that only inverse agonists were able to reduce transcriptional levels below basal and with that the production of pyocyanin. This implies that the aim is to search rather for inverse agonists than for antagonists [71].

### Ligand-based design

**Figure 14:**
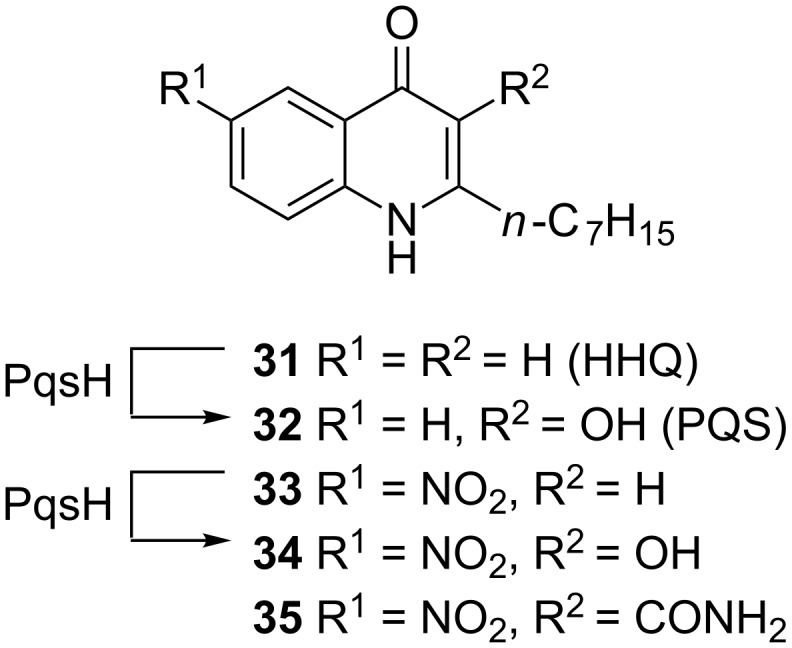
Native PqsR ligand HHQ (**31**) which is converted into PQS (**32**) by PqsH and synthetic inhibitors **33** and **35**, former is also converted by PqsH into the strong agonist **34**.

Following a ligand-based approach Lu et al. modified the native PqsR ligand HHQ (**31**) by introducing a strong electron-withdrawing nitro group in the 6-position (compound **33**) [72]. While displaying an IC_50_ of 51 nM in an *E. coli*-based reporter gene assay, **33** was also able to reduce pyocyanin production to 44% at 15 µM. Further investigations showed that when conducting the reporter gene assay in *P. aeruginosa* instead of using the heterologous *E. coli* system activity of **33** was drastically reduced (only 60% inhibition at 10 µM). The reason for this drop in activity was the cell-mediated oxidation of the 3-position of the quinolone core through action of the *P. aeruginosa* enzyme PqsH (Figure 14), turning the inverse agonist **33** into a strong agonist **34** (EC_50_ = 2.8 nM).

This phenomenon was overcome by blocking the metabolic susceptible 3-position with various functional groups resulting in **35** which showed good activity in both *E. coli* (IC_50_ = 35 nM) and *P. aeruginosa* (IC_50_ = 404 nM) based reporter gene assays. Furthermore, this compound was able to inhibit pyocyanin production with an IC_50_ of 2 µM and HHQ levels were reduced to 54% at a concentration of 15 µM. Additionally the Hartmann group demonstrated that **35** enables survival of PA14-infected *Galleria melonella* larvae [73]. Moreover, the optimised compound also benefited from a decreased clogP value compared to the parent compound **33**, which is reflected in an improved solubility [74]. In a recent publication by Kamal et al. the pharmacological profiles of several alkylquinolone compounds were investigated in a structure–functionality relationship manner, resulting in four different profiles: (a) agonists, (b) antagonists, (c) inverse agonists and (d) biphasic modulators. These studies revealed that pyocyanin production is only decreased significantly when the QS modulators are inverse agonists. It was hypothesized that the already mentioned 3-position is crucial for the functionality. Depending on the groups installed in this position and, hence, the different ligand–protein interactions they introduce, compounds are either agonists, antagonists, or inverse agonists. This hypothesis was in accordance with a study made by Shanahan et al. who synthesized various other C-3 substituted analogs [75].

**Figure 15:**
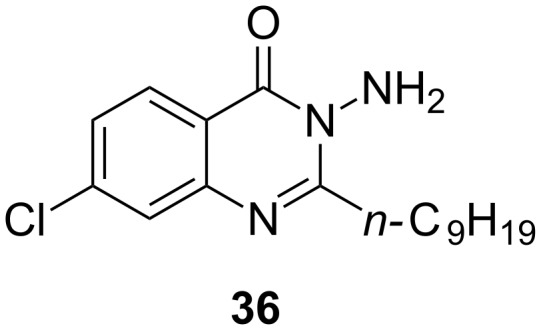
Quinazolinone inhibitor **36** (QZN).

**Figure 16:**
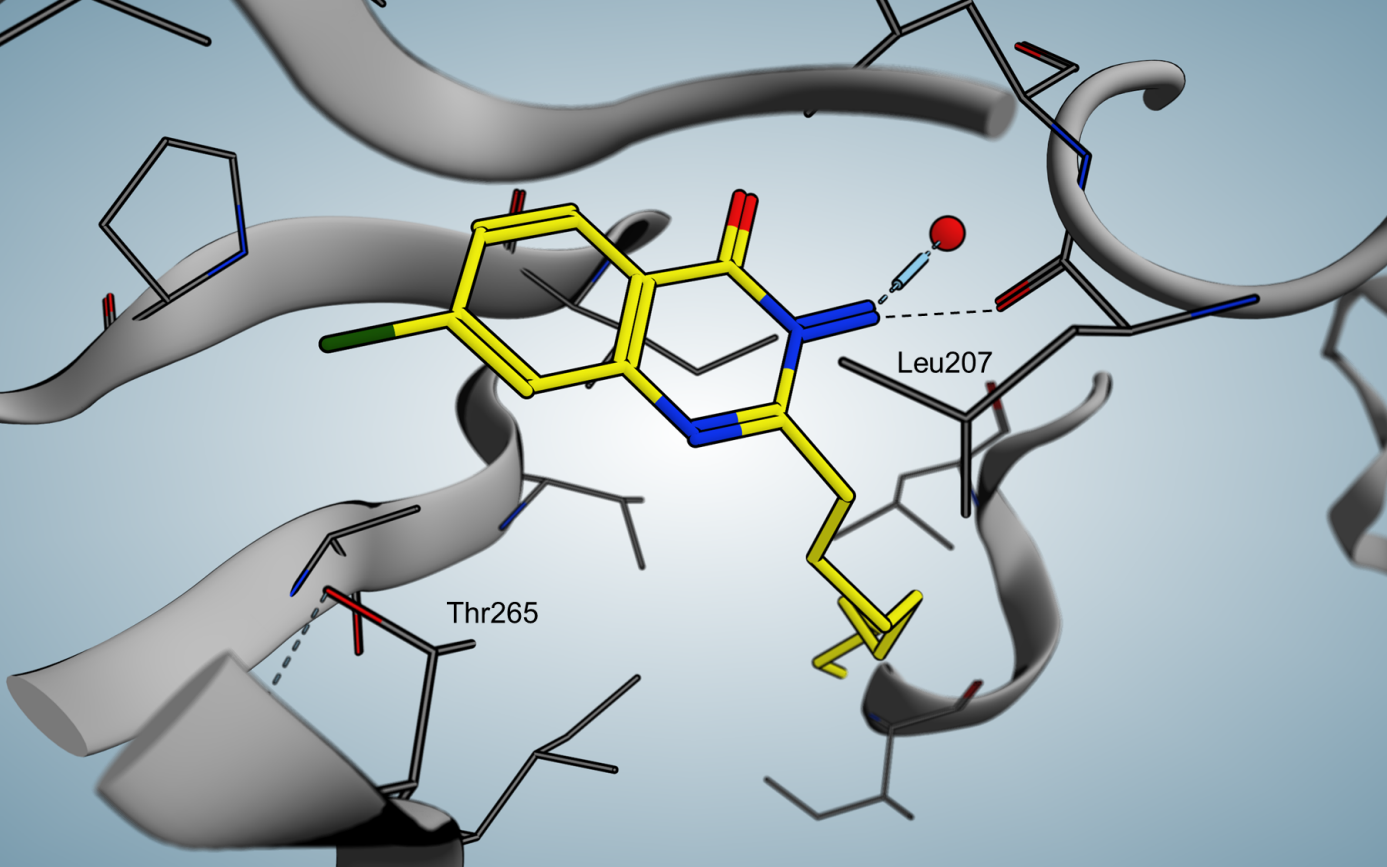
Crystal structure of QZN (**36**) in complex with PqsR^CBD^.

Ilangovan et al. discovered a quinazoline scaffold as another class of ligand-based hit compounds. Based on the C9-congener of HHQ several substituted 2-alkyl-4(*H*)-quinazolines were synthesized. The most potent compound **36** (Figure 15) showed micromolar inhibition in *P. aeruginosa* and was able to decrease pyocyanin levels down to less than 0.5 µg/mL at 100 µM. Furthermore, AQ signal molecules could also be suppressed. The crystal structure of the PqsR co-inducer binding site in complex with **36** was solved at 2.95 Å resolution (Figure 16), as well as a co-crystal structure of the native HHQ C9-congener NHQ [76] demonstrating a competitive binding mode.

When compared to the native ligand NHQ, **36** shows similar hydrophobic interactions (Figure 16). In addition, the chlorine is able to occupy a vacant sub pocket. A hydrogen bond is found between the backbone oxygen of L207 and the 3-NH_2_ hydrogen atoms. Interestingly, adding the chlorine substituent in 7-position of PQS leads to a 135 times more potent agonist, indicating the importance of the vacant sub pocket next to T265. This also indicates that the quorum quenching activity of **36** depends on slight conformational changes. The L1 loop main chain and a rotation of the T265 side chain are hypothesized to be important for antagonistic/inverse agonistic functionality of PqsR-targeting QSIs.

**Figure 17:**
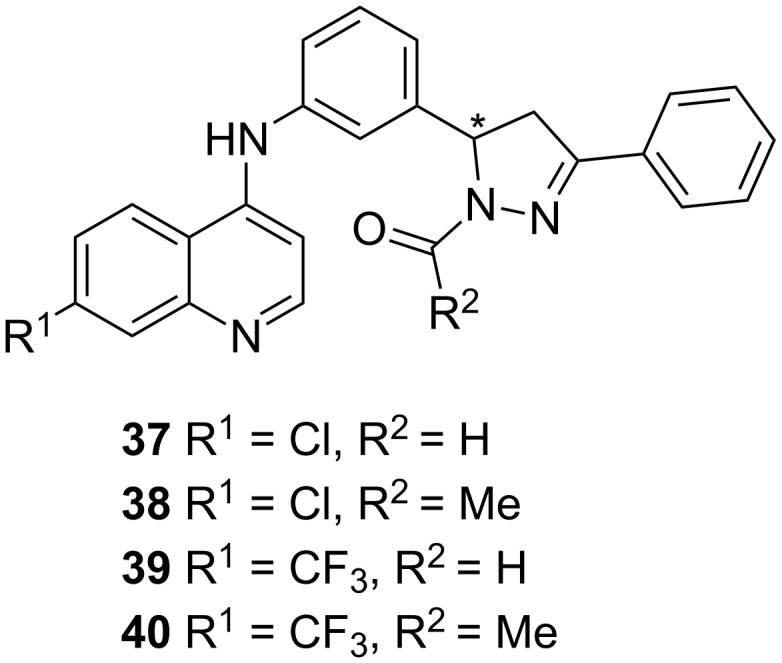
Structures of best fitting compounds **37**–**40** obtained from docking studies.

More recently the same group used docking studies to select compounds from a quinolone-based compound library (Figure 17). The best fitting compounds **37**–**40** were then evaluated in a whole bacterial cell-based *P. aeruginosa* screening with IC_50_ values in the low micromolar range. Additionally, they showed that compound **37** emerged as the most potent inhibitor of this series.

Compounds **37** and **39** furthermore exhibited inhibition of HHQ, PQS and HQNO production in PAO1 strains when treated with 3 × IC_50_, whereas in PA14 a strong decrease in activity could be observed, especially for **39**.

### Benzamide-benzimidazole (BB) series

**Figure 18:**
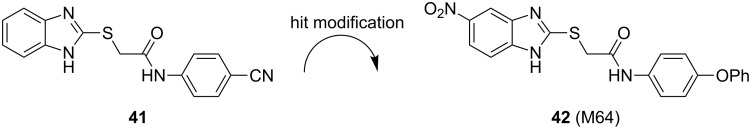
Initial hit **21** and optimized compound **42** (M64).

In 2014, Starkey et al. performed a high-throughput whole-cell screening and identified the benzamide-benzimidazole (BB) motif as a promising scaffold for the inhibition of PqsR [77]. Starting from **41**, which was not only able to suppress expression of AQs but also completely blocked pyocyanin production at a concentration of 10 µM. Various analogues were synthesized resulting in compound **42** (M64), where similar as in the quinolones described by Lu et al., introduction of the electron-withdrawing nitro function led to very potent inverse agonist (Figure 18) [72]. M64 (**42**) proved a very potent inhibitor of HAQ and pyocyanin production at 1 µM.

**Figure 19:**
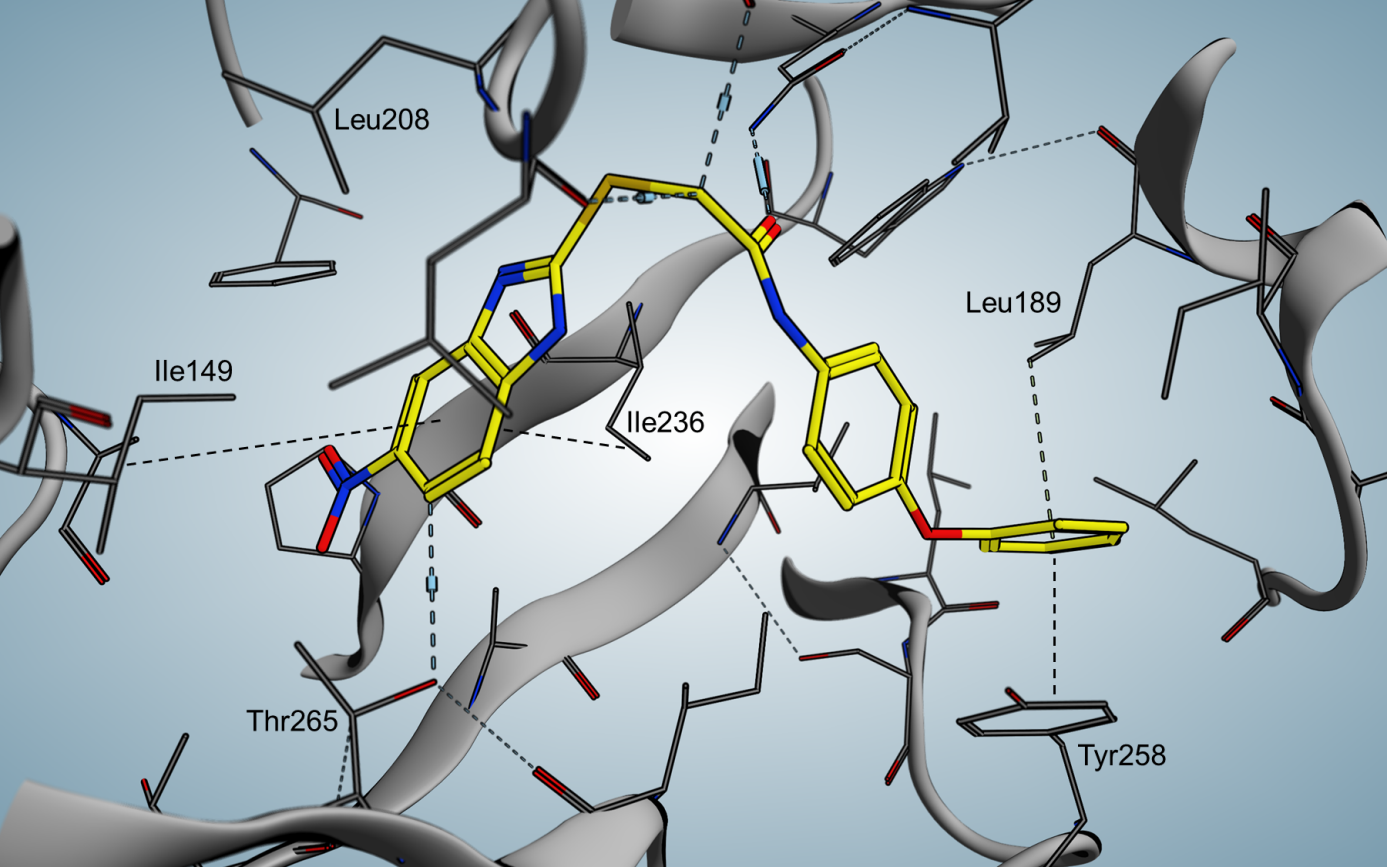
Co-crystal structure of M64 (**42**) with PqsR^LBD^.

Further investigations revealed that M64 (**42**) also reduces 2-AA levels leading to a decreased rate of persister cells. The compound also proved to be active in burn wound and lung infection models in mice and increased survival rates especially when applied in combination with sub-therapeutic doses of ciprofloxacin. In an analytics-driven study by Allegretta et al., the compound was further profiled regarding suppression of the PQS-related metabolites DHQ, 2-AA, HHQ, PQS and HQNO. In brief, this study demonstrated that PqsR is an excellent target for potent QSI compounds effectively suppressing AQ levels and 2-AA production at reasonable concentrations [69]. Lately, Maura and Rahme were able to demonstrate the effect of M64 (**42**) on biofilm formation [78]. Biofilm biomass was drastically reduced when treated with 1 and 10 µM of **42** compared to the untreated PA14 control. Function of PqsR is involved in regulation of HQNO-mediated autolysis and eDNA release, which has been reported to be important for antibiotic tolerance of biofilm-inheriting bacteria. Hence, Rahme and co-workers investigated the effect of their compound **42** on the ability to improve antibiofilm effects of two clinically relevant drugs. When growing biofilms for 48 h in presence or absence of M64 (**42**), followed by treatment of 10 µg/mL of meropenem or tobramycin for 24 h, the activity of the otherwise ineffective antibiotics could be restored. Especially in the case of meropenem, which did not have any effects at all on biofilm viable cells, **42** lead to remarkable results. In 2018, Kitao et al. solved the crystal structure for PqsR ligand binding domain in complex with M64 (**42**) with a resolution of 2.65 Å (Figure 19), unravelling the exact interactions of the compound with the protein [79].

Indicated key interactions are π-stacking of Y258 with the phenoxy moiety in the tail region and a hydrogen bond formed between the Q194 side chain and the carboxamide in the linker area. Furthermore the benzimidazole core shows hydrophobic contacts with isoleucins 149 and 236. More hydrophobic interactions were observed in the tail region, in particular with leucins 189 and 208 as well as Y258. Mutations at these specific residues indicated that the π–π interactions of Y258 are crucial for M64's full inhibition with respect to pyocyanin production, which was only weakly inhibited in an Y258M *P. aeruginosa* mutant strain. The importance of the phenoxy substitutent was further supported by a congener of M64 that lacks this motif and therefore is unable to be involved in π-stacking resulting in a nine-fold increased IC_50_ value compared to M64. Even though there is no specific interaction observed for the nitro function it is crucial for the activity and thus believed to form an instable H-bond with T265. The Rahme group already demonstrated in a former ITC assay that M64 is directly bound in the PqsR LBD [77]. However, they were also suggesting inverse agonistic effect of M64 based on mutation experiments [79]. Moreover an in vivo cross-linking assay of full-length PqsR and a corresponding I68F mutant was carried out leading to the suggestion that upon binding of M64 the protein stability might be increased. Based on these results it was proposed that M64 induces a change in conformation of the PqsR-DNA binding domain, whereas the LBD is not affected extensively.

### Aryloxyacetindoles

**Figure 20:**
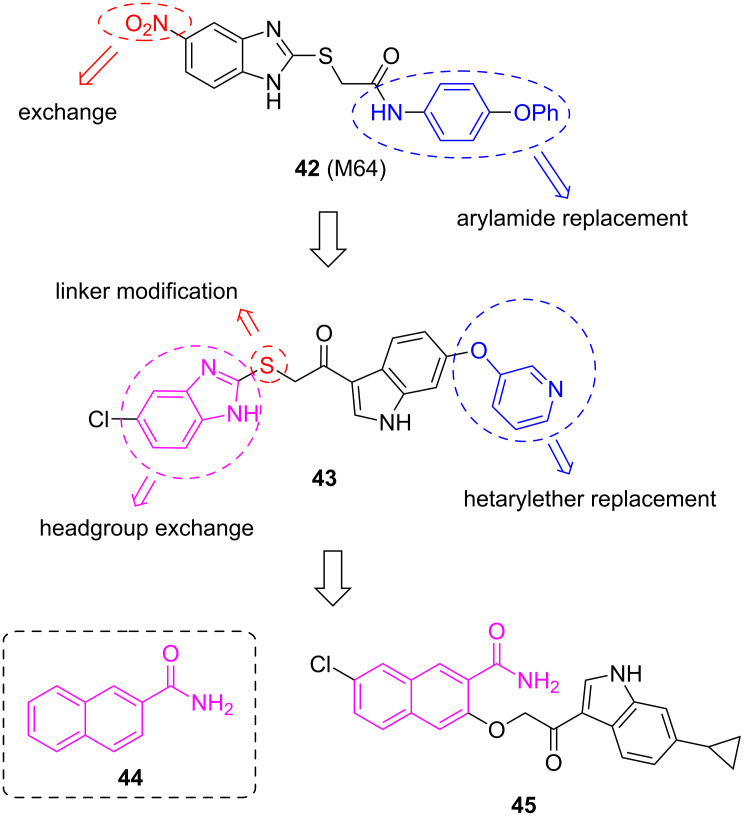
M64 (**42**) as the starting point for further optimization leading to **43**, which was further modified and merged with the fragment **44** to give compound **45**.

Spero Therapeutics further optimized M64 (**42**), firstly by changing the phenoxyphenylamide into a carbonyl-linked indole containing a hetarylether (**43**) [80]. Afterwards they varied the linkage of the benzimidazole moiety (compound not shown) [80]. In a follow-up patent they generated PqsR inhibitor **45** as a front-runner compound [81], in which the benzimidazole headgroup was replaced by a substituted naphthalene bearing a carboxamide, in analogy to a fragment **44** of Zender et al. [82] and similar to the carboxamide-decorated nitro-quinoline scaffold described by Lu et al. (Figure 20) [73].

Compound **45** was highly potent in inhibiting pyocyanin production (stated IC_50_ in a range of 50–250 nM) and was furthermore able to suppress PQS and HHQ production (50 nM < IC_50_ < 250 nM). In a murine thigh infection model using PA14, target engagement was demonstrated in vivo measuring PQS and HHQ levels from the corresponding tissues after treatment. Compound **45** was able to reduce PQS and HHQ levels to 50% and 40%, respectively, 12 hours post-infection. Up to now no further optimization or development of these compounds has been reported.

### Fragment-based design

**Figure 21:**
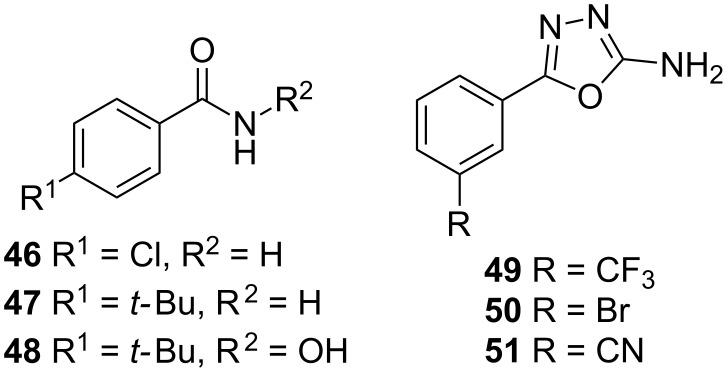
Hit fragments from the benzamide (**47**–**48**) and oxadiazole class (**49**–**51**).

In 2012 Klein et al. obtained the benzamides **46** and **47**, as well as the hydroxamic acid **48** as hits within an SPR screening, which were further evaluated in ITC experiments in order to have a clearer view on the thermodynamic parameters (Figure 21). The antagonists displayed activity in a low double-digit µM range, but had only a marginal impact on the production of the virulence factor pyocyanin [83].

Further SPR screenings afforded hits **49**–**51** with EC_50_ between 7.5–17.8 µM. When compared to the benzamide class, compound **49** shows no significant increase in affinity to the target receptor but is able to inhibit pycocyanin formation by 46 ± 9% at a concentration of 250 µM, and is capable of reducing the AQ’s HHQ and HQNO up to 43 ± 3% at the same concentration [82]. With these fragments in hand further growing and subsequent linking or merging may open new avenues for the generation of new drug-like PqsR inverse agonists.

### Dual target QS inhibitors

#### PqsBC/PqsR

**Figure 22:**
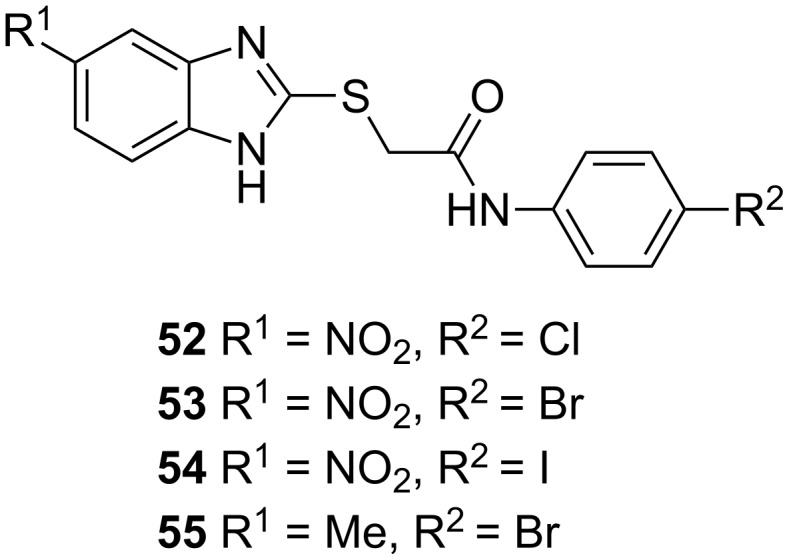
Structures of dual inhibitors **52**–**55**.

In an initial target assessment, Maura et al. found that compound **52** showed an ambiguous profile. This raised the question if this compound class could target additional targets of the PQS-system besides PqsR [68]. Experiments with a PqsR isogenic mutant strain revealed that **52** inhibits HHQ and PQS production, while raising 2-AA levels, pointing at PqsBC as a second target, which was corroborated via SPR studies. When exchanging the chlorine to bromine **53** a high PqsR activity was obtained while the affinity to PqsBC decreased (Figure 22).

The iodine-substituted derivative **54** showed both, a high PqsR, as well as a high PqsBC activity. Exchanging the electron-withdrawing nitro functionality with an electron-donating methyl group turned the PqsR antagonist **53** into a very potent PqsBC inhibitor while losing activity on the initial target PqsR. In addition to these mechanistic findings, it was also shown that the dual inhibitors are capable of rescuing human lung epithelial cells and macrophages at a concentration of 50 µM in cell-based infection models. Also antibiotic activity of meropenem (dose: 10 µg/mL) in presence of 10 µM of dual inhibitors could be partially reinstalled.

#### PqsD/PqsR

**Figure 23:**
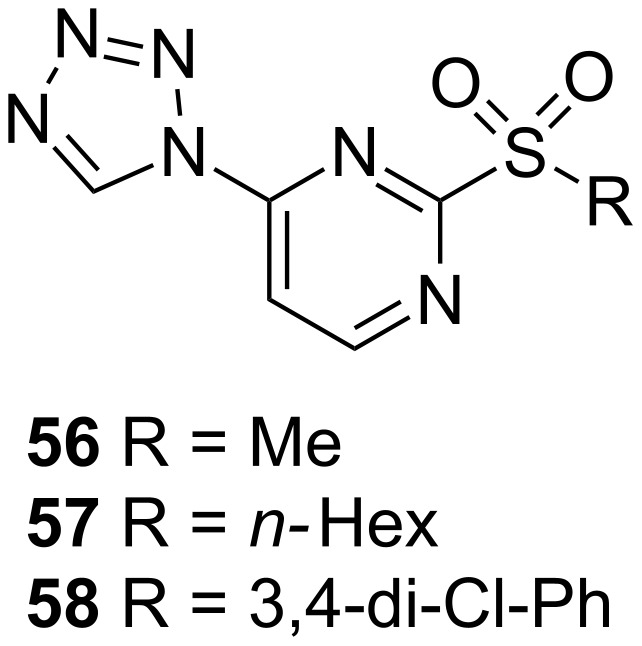
Sulfonyl pyrimidines **56**–**58** acting as dual PqsD/PqsR inhibitors.

Thomann et al. showed that combining a PqsD and a PqsR activity synergistically affects pyocyanin production. Based on these results combining fragments from a PqsR and a PqsD inhibitor belonging to a sulfonyl-pyrimidine class, compound **56** was generated and its ability to reduce pyocyanin evaluated (Figure 23) [84]. While exhibiting IC_50_ values of 15 µM on PqsR and 21 µM on PqsD, the compound was able to inhibit the pyocyanin production with an IC_50_ of 86 µM. Moreover **56** also proved to be efficient in blocking pyoverdine production, another important *P.aeruginosa* virulence factor. When applied at a concentration of 500 µM less than 10% of pyoverdine production was remaining. At 100 µM the pyoverdine amount was cut to a half. Since also the levels of extracellular DNA could be reduced to a minimum with their dual inhibitor, the group investigated the effect of adding **56** to ciprofloxacin. The combination of this QSI together with the antibiotic significantly increased antibiofilm activity at the used concentrations ([CIPX] = 1 µM, [56] = 50 µM). The compound also proved to be active in a *Galleria melonella* survival assay being capable of ensuring survival up to more than 50% after 4.5 d post-infection at a dosage of 1.25 nmol compared to untreated PA14-infected larvae.

Following the dual inhibitor concept, the class of sulfonylpyrimidines further afforded compounds **57** and **58** with promising activity. While PqsR activity slightly decreased (50 µM and 24 ± 5 µM, respectively) **57** exhibited an IC_50_ of 1.7 µM and **58** displayed sub-micromolar activity of 0.4 ± 0.1 µM. The effects on biofilm formation and eDNA release were evaluated at a concentration of 100 µM. Even if **57** was less potent on both PqsR and PqsD compared to **58**, it turned out to be more efficient in inhibiting biofilm production. When assessed on their ability to reduce extracellular DNA all three compounds were equally potent. Nevertheless compound **58** only showed a weak effect on the inhibition of pyocyanin (14% inhibition at 100 µM) [85].

## Conclusion

In the past decade, the pqs QS system of *P. aeruginosa* has attracted increasing interest by academic researchers. This is certainly due to its prominent involvement in virulence regulation of this important Gram-negative pathogen. Among the various pathoblocker strategies, targeting a master regulator of pathogenicity traits appears to have huge translational potential. Hitting an array of virulence mechanisms at once instead of addressing just singular factor holds great promise for future discovery and development of pqs-targeting QSI. Compared to the other QS systems present in *P. aeruginosa* the pqs system is lacking some of the liabilities associated with the las and the rhl systems. The former AHL-dependent regulatory circuit has been described to be the first QS system to get lost upon chronification of *P. aeruginosa* infections [86]. However, chronic lung infections are one of the major indications with a very high medical need. In the case of addressing the rhl system, a non-unidirectional virulence modulatory effect is observed. Agonists of RhlR reduce pyocyanin, but induce rhamnolipid production, while antagonists have the inverted effect [87]. This raises some concerns about the applicability of RhlR as an effective ‘stand-alone’ pathoblocker target. A combination of rhl- and pqs-targeting QSI, however, seemed to provide promising and clear-cut antivirulence effects [88]. Finally, the potential of iqs-targeting approaches remains to be investigated as more insight in the function of this rather recently discovered regulator is needed. The pqs system is active in chronically infected cystic fibrosis patients and, according to the current knowledge, blockade of this master regulator delivers an unambiguous antivirulence effect.

In terms of published research, the most studied molecular targets within the pqs system are the signal molecule synthase PqsD and the receptor PqsR (MvfR), while in the latter case projects are currently in a clearly more advanced stage. When comparing the reported antivirulence effects of PqsD- and PqsR-targeting QSI, evidence is growing that hitting the transcriptional regulator results in more pronounced pathoblocking effects than addressing the biosynthetic enzyme cascade. However, a synergistic effect for dual-target inhibitors hitting PqsR and PqsD or PqsBC simultaneously has been described [68,84]. Additionally, the authors believe that attempts to effectively target PqsE are still worthwhile pursuing, given its prominent involvement in pqs virulence regulation. However, this would require elucidating the still unknown mechanism behind its regulatory function.

In order to translate the promising hit and lead compounds described above into the clinic, continuous discovery and development efforts are required. Especially the lead optimization stage is strongly dependent on integrated medicinal chemistry and biological profiling teams. In addition to potency considerations, drug-like properties aiming at favorable pharmacokinetics move into the focus [89]. Due to the complex nature of virulence phenotype assays as well as ADME/T testing cascades assembling the required teams, expertise, and resources might be a challenge especially for academic groups. Hence, often proclaimed drug discovery timelines for target-to-candidate projects of about 6 years or less [90] are quite unrealistic in this field. This actually underpins the urgency for current anti-infective discovery efforts to enable refilling the pipeline in due time before available treatment options run out. However, we believe the translational perspective for such pathoblockers is quite promising. Specifically, it has been shown that PqsR-targeting QSI are able to increase the susceptibility of *P. aeruginosa* biofilms against antibiotics [78]. Hence, adjunctive treatment approaches where a conventional backbone antibiotic therapy is potentiated by pathoblocking agents appears quite attractive. In analogy to current antiviral and anticancer strategies, more personalized pathogen-specific drug combinations should be pursued also in the bacterial infections field. As a consequence, more advanced diagnostic tools have to be devised to enable fast and reliable analysis of the pathogen and its resistance profile. We are curious what future research will uncover in this important, yet underexploited, drug discovery field and believe exploring such strategies further will be a worthwhile endeavour.
